# Novel Amdoparvovirus Infecting Farmed Raccoon Dogs and Arctic Foxes

**DOI:** 10.3201/eid2012.140289

**Published:** 2014-12

**Authors:** Xi-Qun Shao, Yong-Jun Wen, Heng-Xing Ba, Xiu-Ting Zhang, Zhi-Gang Yue, Ke-Jian Wang, Chun-Yi Li, Jianming Qiu, Fu-He Yang

**Affiliations:** State Key Laboratory for Molecular Biology of Special Economical Animals, Institute of Special Animal and Plant Sciences, Chinese Academy of Agricultural Sciences, Changchun, China (X.Q. Shao, Y.J. Wen, H.X. Ba, X.T. Zhang, Z.G. Yue, C.Y. Li, F.H. Yang);; State Key Laboratory of Marine Environmental Science, Xiamen University, Xiamen, China (K.J. Wang);; University of Kansas Medical Center, Kansas City, Kansas, USA (J Qiu)

**Keywords:** amdoparvovirus, infection, raccoon dog (Nyctereutes procyonoides), Arctic fox (Vulpes lagopus), viruses, China

## Abstract

A new amdoparvovirus, named raccoon dog and fox amdoparvovirus (RFAV), was identified in farmed sick raccoon dogs and arctic foxes. Phylogenetic analyses showed that RFAV belongs to a new species within the genus *Amdoparvovirus* of the family *Parvoviridae*. An RFAV strain was isolated in Crandell feline kidney cell culture.

Amdoparvoviruses, members of the autonomous parvoviruses, belong to the family *Parvoviridae*, subfamily *Parvovirinae*, genus *Amdoparvovirus*(*1*). Only 2 distant species have been reported: *Carnivore amdoparvovirus 1*, which comprises only Aleutian mink disease virus (AMDV), and *C. amdoparvovirus 2*, which comprises only gray fox amdovirus (*1*,*2*). Natural AMDV infection mainly occurs in the *Mustelidae* family (*3*) and causes immune complex–mediated disease (*4*). However, to our knowledge, natural amdoparvovirus infection in raccoon dogs or arctic foxes has not been reported. We describe the identification, isolation, and infection of a novel amdoparvovirus in canids, which represents a new viral species (*C. amdoparvovirus 3*), named raccoon dog and fox amdoparvovirus (RFAV), within the *Amdoparvovirus* genus.

## The Study

During July–December 2012 and 2013, sick raccoon dogs and arctic foxes, which were farmed for fur products on 6 farms (farms A–F) in Jilin and Liaoning provinces, China, were received for quarantine inspection at the Fur Animal Disease Laboratory, Institute of Special Animal and Plant Sciences, Chinese Academy of Agricultural Sciences. Several infant raccoon dogs from 1 litter became ill 40 days after birth, and the numbers of sick animals increased by the time they were 3 months of age. Clinical signs included anorexia, emaciation, growth retardation, thirst, chronic diarrhea, and unkempt fur; necropsy often revealed cyanosed splenomegaly, enlargement of mesenteric lymph nodes, and renal cortex congestion and brittleness. For the raccoon dogs showing similar clinical signs, rate of illness was 4%–8%; death rate was ≈60% before the age of 4 months; and rate of illness increased by years on the farms that initially had sick animals. Among arctic foxes, signs varied: emaciation and growth retardation in 3-month-old cubs with pale and swelling kidneys in dead foxes; and severe diarrhea or intermittent tar-like feces in 3–7-month-old cubs. Antibacterial drug treatment was ineffective in these diseased animals.

Because signs in the sick animals sent for quarantine inspection were similar to those in Aleutian mink disease, we first used AMDV-specific counter-immunoelectrophoresis (CIEP) (*5*) to test serum samples of six 3-month-old sick raccoon dogs from farm A. All 6 were positive. Next, we designed conserved amdoparvovirus primers (AV7; Table 1) for PCR detection. Viral nucleic acids were extracted by using a MiniBest Viral RNA/DNA Extraction Kit (TaKaRa, Dalian, China). DNA extracted from spleen, kidney, mesenteric lymphonodus, and mucosal tissue and blood of the 4 sick raccoon dogs was all RFAV DNA positive. After 10 days, the 4 raccoon dogs remained RFAV DNA PCR positive in blood and CIEP positive in serum. DNA extracted from blood of two 3–7-month-old sick raccoon dogs from farm C was RFAV DNA PCR positive, and serum samples from these animals were CIEP positive. Two 7-month-old raccoon dogs from farm D appeared healthy but on necropsy showed cyanosed splenomegaly. Their blood and spleens were RFAV DNA positive, and serum was CIEP positive. The overall positive rates of RFAV DNA and CEIP antibody in sick raccoon dogs were 90% and 100%, respectively.

**Table 1:** Oligonucleotide primer pairs used for PCR amplification of amdoparvovirus

Gene, name	Primer (5’ → 3’)	Ta,† amplicon	Use
VP2			
AV7-F	CCAACAAGTAATGACACCTTGGT	52°C	Detection
AV7-R	CCTGCTGGTATTATCCATTCAGGA	≈786 bp	Sequencing
AV3-F	CCAACAAGTAATGACACCTTGGT	53°C	Detection
AV3-R	GGTTGGTTTGGTTGCTCTCCAAGGA	316 bp	
NS1			
ANS-F	GTAACATGGCTCAGGCTCA	52°C	
ANS-R	CTCATGCCGAGGTCTCTTGTG	2,008 bp	Sequencing
VP1			
AVP-F1	CACAAGAGACCTCGGCATGAGTA	52°C	
AVP-R1	CCTGCTGGTATTATCCATTCAGGA	≈1,297 bp	Sequencing
AVP-F2	GGCTTTGTTCCTTGGAGAGCAAC	52°C	
AVP-R2	TGGTAGAATRAGGAAGTACACAK	≈1,443 bp	Sequencing

*NS, nonstructural protein; VP, viral structural protein. †Annealing temperature.

Serum iodine agglutination test (IAT) (*6*) was positive or strongly positive in 20 sick raccoon dogs that were 7 and 19 months old but was negative in healthy animals. Of the 29 sick raccoon dogs, two 3–7-month-old animals were both canine parvovirus 2 and RFAV DNA positive by PCR in blood or spleen samples. However, canine distemper virus was not detected in spleen samples from any sick raccoon dogs.

In arctic foxes raised together with sick raccoon dogs on farm B, intestinal mucosa samples from 3 of 7 foxes that died of diarrhea-associated dehydration were RFAV DNA PCR positive. One kidney sample from a 3-month-old fox of 3 tested on farm F showed renal enlargement and was RFAV DNA PCR positive. Blood, urine, and feces of two 7-month-old arctic foxes on farm C, which had tar-like feces, were RFAV DNA PCR positive. Serum samples of these 2 foxes were CIEP positive. Both PCR and CIEP remained positive for at least 2 months. Results of all samples tested by PCR, CIEP, and IAT are summarized in Table 2.

**Table 2:** Detection of raccoon dog and fox amdoparvovirus in sick and healthy animals using PCR, CIEP and IAT, China*

Animal, health status	Age, mo.	Year	Farm†	No.	Rate			
					PCR+‡	CIEP+	IAT+	CIEP+PCR+
Raccoon dog								
Diseased	3-7		Total	10	8/10	8/8	2/2	6/8
		2012	A	6	4/6	6/6	ND	4/6
		2012	B	2	2/2	ND	ND	NA
		2013	C	2	2/2	2/2	2/2	2/2
	7, 19		Total	18, 1	18/19	18/18	18/18	17/18
		2013	D	12, 1	12/13	13/13	13/13	12/13
		2012	A	5	5/5	5/5	5/5	5/5
		2012	E	1	1/1	ND	ND	NA
Healthy§	3	2013	Total	10	0/10	0/10	ND	0/10
	7	2013	Total	5	0/5	2/5	0/5	0/5
Arctic fox								
Diseased	3		Total	10	4/10	ND	ND	NA
		2012	B	7	3/7	ND	ND	NA
		2012	F	3	1/3	ND	ND	NA
	7–9	2012	C	2	2/2	2/2	ND	2/2
Healthy	7	2012	Total	5	0/5	0/5	0/5	0/5

*CIEP, counter-immunoelectrophoresis; IAT, serum iodine agglutination test; NA, samples not available; ND, assays not done; +, positive. †Farm: A, Heishan County; B, Qian’an County; C, Jilin County; D, Panjin County; E, Haicheng County; F, Nong’an County. Qian’an, Jilin, and Nong’an are located in Jilin province; Heishan, Panjin and Haicheng are located in Liaoning province. ‡For raccoon dogs, PCR+ for all the tested tissues (blood, spleen, kidney and mesenteric lymphonodus). For arctic foxes, PCR+ results only for 1 of the tested tissues (blood, kidney, or intestinal mucosae). §Blood and spleen samples were tested separately by PCR from healthy animals except for the blood samples from the 3-month-old healthy raccoon dogs.

We next applied a semiquantitative PCR to quantify the level of RFAV DNA in the blood and spleens of four 7-month-old sick raccoon dogs (3 from farm D, 1 from farm C). Briefly, DNA samples were diluted from 10^1^ to 10^7^ viral genomic copies (vgc)/μL by using a quantified RFAV DNA template and were amplified by using AV3 primers with the detection threshold of ≈100 vgc in a volume of 15 μL. Virus titer was determined on the basis of the maximum dilution at which viral DNA was detected by agarose gel electrophoresis. RFAV DNA ranged from ≈2 × 10^5^ to ≈5 × 10^7^ vgc/mL in blood and ≈7 × 10^7^ vgc/g in spleen. In spleen and kidney tissues of 2 raccoon dogs euthanized at 3 months of age (from farm A) and four 7-month-old sick raccoon dogs (from farm D), bacterial infections, performed by standard methods, were not found. Collectively, these results demonstrate that blood or tissues from the 2 sick animals contained RFAV DNA, and the DNA levels were high in some samples.

**Figure 1:**
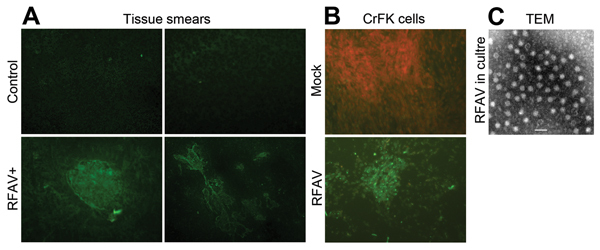
Detection of amdoparvovirus antigens in sick raccoon dogs and infected cells. A) Detection of amdoparvovirus antigens in tissues of sick raccoon dogs. Tissue smears, as described below, were prepared from spleen and kidney samples of sick raccoon dogs and detected with a control mouse serum or anti-AMDV-G serum by using an IFA (*8*). (a) Mock smear of a spleen tissue from a sick raccoon dog, detected with normal mouse serum as a primary antibody; (b) Control smear of a spleen tissue from a healthy raccoon dog; (c,d) amdoparvovirus antigen-positive smears of spleen tissue (c) and kidney tissue (d) from a sick raccoon dog, detected with anti-AMDV-G serum. Original magnification ×200. B,C) Detection of amdoparvovirus antigens and virions in infected CrFK cells. B) One milliliter filtered (0.22 μm) pathological spleen samples collected from a sick raccoon dog that had a virus titer of ≈7× 10^7^ vgc/mL, was inoculated to confluent CrFK cells in a T25 flask. Infected cells of the fourth passage were fixed and analyzed with IFA by using anti-AMDV serum (mock- or RFAV-infected CrFK cells). C) Twenty-five milliliter supernatant (≈2× 10^9^ vgc/mL) of RFAV(XQ-JLR)–infected CrFK cell cultures were concentrated. Virions were agglutinated with an anti-RFAV raccoon dog serum and were visualized under a transmission electron microscope (TEM). AMDV, Aleutian mink disease virus; RFAV, raccoon dog and fox amdoparvovirus; IFA, indirect immunofluorescence assay; vgc, viral genome copies. Scale bar = 50 nm.

We used indirect immunofluorescence assay (IFA) to probe RFAV antigens in spleen and kidney of the 2 RFAV PCR–positive 3-month-old sick raccoon dogs from farm A. Tissues of the sick raccoon dogs were AMDV-G antigen IFA positive, but the healthy animal tissues were not (Figure 1, panel A). This result indicates that the spleen and kidney of the sick raccoon dogs contained viral antigens that share immunogenicity with AMDV-G. More importantly, we isolated an RFAV strain, named XQ-JLR, by infecting CrFK cells. IFA using anti-AMDV serum showed positive green cells in RFAV-infected CrFK cells but not in mock-infected cells (Figure 1, panel B). Under a transmission electron microscope, virus particles at ≈23 nm in diameter were visualized in a concentrated supernatant of infected CrFK cells (Figure 1, panel C).

We further proved that RFAV is the predominant virus in the lesion tissues of sick animals. A modified SISPA (*7*) was performed for high-throughput sequencing with mixed lesion tissues of 3 spleens and 3 kidneys from 3 sick raccoon dogs. By using Illumina MiSeq sequencing (Illumina, San Diego, CA, USA), we obtained 478,813 high-quality reads, and 668 contigs were assembled, including 17 contigs for new amdoparvovirus. Amdoparvovirus sequences, except for a difficult-to-sequence high guanine–cytosine nucleotide content of 62-bp gap, were recovered, which are consistent with the sequences acquired by Sanger sequencing of viral DNA amplified from tissues. There were 865 reads, by BLASTn (http://www.blast.ncbi.nlm.nih.gov/Blast.cgi), in alignment with amdoparvovirus sequences but only 16 reads with sequences of other non-mammal viruses, such as phage and baculovirus. The identities were <91% of the reads aligning with AMDV sequences in GenBank by BLASTn (E value <10^−5^).

**Figure 2:**
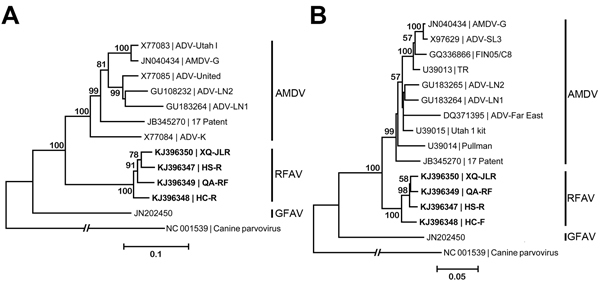
Phylogenetic analyses of amdoparvoviruses. A) Phylogenetic tree based on the viral NS1 gene. B) A phylogenetic tree based on the major capsid VP2. RFAV and other published amdoparvovirus sequences were aligned by using the MUSCLE program in MEGA5.2 (*9*), which used a P-distance model with 1,000 bootstrap replicates to generate phylogenetic trees of NS1 and VP2 aa sequences. GenBank accession numbers of isolates or strains are shown on the tree. Canine parvovirus was used as an outgroup. AMDV, Aleutian mink disease virus; GFAV, gray fox amdoparvovirus; RFAV, raccoon dog and fox amdoparvovirus; NS, nonstructural protein; VP, viral structural protein. Sequences obtained from this study are shown in bold. Scale bars indicate nucleotide substitutions per site.

We sequenced AV7 primer-amplified PCR products of RFAV DNA from the tissues of 32 animals (GenBank accession nos. KJ396347– KJ396358). Four representative strains of nearly full-length genome sequences (GenBank accession nos. KJ396347–KJ396350) only share a similarity of 82% and 76.7% in the nonstructural protein (NS) 1–encoding sequence and NS1 aa sequence, respectively, with AMDV. Phylogenetic analyses of 2 neighbor-joining trees of either NS1 or major structural protein (VP2) strongly suggest that RFAV strains cluster into a unique clade between AMDV and gray fox amdovirus species (Figure 2).

## Conclusions

We identified a new virus species, RFAV, from farmed raccoon dogs and arctic foxes in Jilin and Liaoning provinces, China. Raccoon dogs are naturally susceptible to RFAV infection, and RFAV is most likely the etiologic agent responsible for the disease manifestations of the sick raccoon dogs.

## References

[1] Cotmore SF, Agbandje-McKenna M, Chiorini JA, Mukha DV, Pintel DJ, Qiu J, The family *Parvoviridae.* Arch Virol. 2014;159:1239–47. 10.1007/s00705-013-1914-1 24212889 PMC4013247

[2] Li L, Pesavento PA, Woods L, Clifford DL, Luff J, Wang C, Novel amdovirus in gray foxes. Emerg Infect Dis. 2011;17:1876–8. 10.3201/eid1710.110233 22000359 PMC3310670

[3] Farid AH. Aleutian mink disease virus in furbearing mammals in Nova Scotia, Canada. Acta Vet Scand. 2013;55:10–55. 10.1186/1751-0147-55-10 23394546 PMC3602201

[4] Porter DD, Larsen AE, Porter HG. The pathogenesis of Aleutian disease of mink. 3. Immune complex arteritis. Am J Pathol. 1973;71:331–44 . 4576760 PMC1903963

[5] Cho HJ, Greenfield J. Eradication of Aleutian disease of mink by eliminating positive counterimmunoelectrophoresis test reactors. J Clin Microbiol. 1978;7:18–22 . 203601 10.1128/jcm.7.1.18-22.1978 PMC274849

[6] Henson JB, Gorham JR, Leader RW. A field test for Aleutian disease. Natl.Fur.News. 1962;34:8–9.

[7] Djikeng A, Halpin R, Kuzmickas R, Depasse J, Feldblyum J, Sengamalay N, Viral genome sequencing by random priming methods. BMC Genomics. 2008;9:5–9. 10.1186/1471-2164-9-5 18179705 PMC2254600

[8] Oleksiewicz MB, Costello F, Huhtanen M, Wolfinbarger JB, Alexandersen S, Bloom ME. Subcellular localization of Aleutian mink disease parvovirus proteins and DNA during permissive infection of Crandell feline kidney cells. J Virol. 1996;70:3242–7 . 8627805 10.1128/jvi.70.5.3242-3247.1996 PMC190188

[9] Tamura K, Peterson D, Peterson N, Stecher G, Nei M, Kumar S. MEGA5: molecular evolutionary genetics analysis using maximum likelihood, evolutionary distance, and maximum parsimony methods. Mol Biol Evol. 2011;28:2731–9 . 10.1093/molbev/msr121 21546353 PMC3203626

